# Livelihood dynamics and challenges to wellbeing in the drylands of rural East Africa – the Drylands Transform study population in the Karamoja border region

**DOI:** 10.1080/16549716.2025.2490330

**Published:** 2025-04-24

**Authors:** Barbara Schumann, Alice Turinawe, Kristina Lindvall, Joseph Lule Kyanjo, Derick Ansyijar Kuule, Caroline Kawira, Annrose Mwangi, Peter Mwangi, Agneta Hörnell

**Affiliations:** a Department of Health and Caring Sciences, Linnaeus University, Kalmar, Sweden; b Department of Agribusiness and Natural Resource Economics, College of Agricultural and Environmental Sciences (CAES) Makerere University, Kampala, Uganda; c Department of Epidemiology and Global Health, Umeå University, Umeå, Sweden; d Department of Environmental Management, School of Forestry, Environmental and Geographical Science (SFEGS) College of Agricultural and Environmental Sciences (CAES), Makerere University, Kampala, Uganda; e Department of Food Science, Nutrition and Technology, University of Nairobi, Nairobi, Kenya; f Department of Food, Nutrition and Culinary Science, Umeå University, Umeå, Sweden; g Department of Forest Ecology and Management, SLU, Umeå, Sweden; h Department of Land Resource Management and Agricultural Technology, University of Nairobi, Nairobi, Kenya

**Keywords:** Kenya, Uganda, agro-pastoralism, malnutrition, sustainable development

## Abstract

**Background:**

The Karamoja region in the East African drylands is a rural, impoverished setting where pastoralism is increasingly replaced by other livelihood strategies. Understanding the socioeconomic contexts as well as their local variations is key for sustainable development of communities.

**Objective:**

The aim of the present paper is to describe the baseline survey of the Drylands Transform project, its setting, methods and key findings.

**Methods:**

In June 2022, a survey was conducted with 944 randomly selected households at four study sites in the Karamoja border region of Kenya and Uganda. Data were analyzed using descriptive statistics.

**Results:**

Main livelihood forms were pastoralism and agropastoralism, while many households also relied on other sources of income. At some study sites, livestock keeping was abandoned by many residents due to cattle raiding and droughts. Only 4% of households were rated as food secure. The proportion of malnutrition among children aged 6–59 months varied across sites between 3% and 17% and was considerably higher among women.

**Conclusions:**

Climate change, water shortage, social conflicts and marginalization pose barriers to food security and wellbeing for rural populations in the East African drylands. There are, however, opportunities for development through income diversification, the improvement of land health, the promotion of kitchen gardens and other measures of sustainable agriculture.

## Paper Context

- **Main findings**: Pastoralist and agropastoralist communities in the Karamoja border region between Kenya and Uganda face high levels of poverty, food insecurity and malnutrition.
- **Added knowledge**: There are considerable variations in malnutrition and other challenges for wellbeing between sites in the study region.
- **Global health impact for policy and action**: Income diversification and improvement of kitchen gardens can contribute to food security, women empowerment and sustainable development in such impoverished rural areas.

## Background

**Figure 1.**
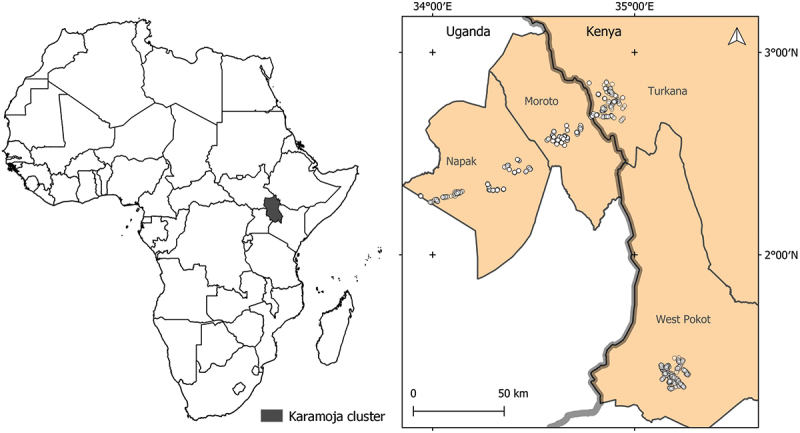
Map of study area. Left: Africa with Karamoja cluster. Right: study sites in the Karamoja border region of Uganda and Kenya. Source: Aida Bargues Tobella, SLU.

East Africa is home to about 6% of the world’s population and over 77% of this population live in rural areas [1]. Their main livelihood is centered around rain-fed agriculture and small-scale business, and therefore, major threats to their livelihood stem from challenges to the agricultural sector. Within East Africa, the Karamoja cluster is located in the border region of north-west Kenya, north-east Uganda, South Sudan and south-west Ethiopia [2]. For the rest of this article, we will solely refer to the border region between Kenya and Uganda as the ‘Karamoja border region’ (Figure 1).

The major activity in this region is livestock keeping. The area is predominantly dryland, receiving limited rainfall and characterized by arid and semi-arid landscapes. It is therefore highly vulnerable to climate change-related impacts such as rising temperature, droughts, erratic and unpredictable rainfall patterns, floods, land degradation and desertification [3–6].

Poverty levels in the Karamoja border region are typically higher than national averages, with some of the lowest social development indicators due to climate change, conflicts and insecurity, and lack of access to infrastructure and essential services [7,8]. Frequent conflicts occur both within and between communities and tribes and may come from neighboring countries. Conflicts tend to arise from scarce natural resources, mostly water and grazing land [5,9], and are exacerbated by climate change [5,10]. They disrupt livelihood activities and displace communities, leading to the loss of livestock and property and derailing development efforts in the whole Karamoja cluster [9,11,12].

In the Karamoja border region of Kenya and Uganda, a large proportion of the population is food insecure due to climatic and socio-economic conditions, and the prevalence of malnutrition is the highest of all Kenyan and Ugandan regions [13–15]. In addition, the communities often face limited access to essential services such as healthcare, clean water and sanitation, and education and skills development. The border region is also characterized by weak infrastructure with poor road networks and inadequate transportation systems, lack of water supply systems, and poor access to markets and financial services [13,16].

Due to these increasing challenges to their livelihoods, communities have been gradually shifting from traditional pastoralism towards a sedentary lifestyle, including diversified activities such as agro-pastoralism, small-scale businesses, and wage labor [16–18].

The Dryland Transform research project is conducted in the Karamoja border region of northeastern Uganda and northwestern Kenya [19]. Its main objective is to investigate the interlinkages between land health, livestock-based livelihoods, human wellbeing, and land governance mechanisms. By this, the project aims to contribute new knowledge for transformative change and sustainable development of socioecological systems in rural drylands of East Africa. The findings from this project are expected to help inform interventions and strategies to improve the livelihoods and overall wellbeing of the population in the Karamoja border region [19–21].

The Drylands Transform project has five objectives, of which the present paper relates to the third objective: ‘Understand the impact of climate variability on livelihood strategies and resilience’ [22]. The present paper addresses the baseline household survey conducted for the third objective. Aims are to a) present survey methods, and to discuss methodological challenges in such settings, and b) describe key findings, i.e. socio-economic characteristics of households and challenges to human wellbeing, as well as their variations across study sites.

## Material and methods

### Study design and setting

Based on a longitudinal panel design, two cross-sectional studies were conducted with randomly selected households to capture livelihoods and wellbeing during two different seasons. Here, we present the design and results of the first survey, conducted from late May to June 2022. Questions were largely identical in the first and the second survey, which was conducted between February and April 2023 with the same household study population.

The study was conducted in the Karamoja border region of northeastern Uganda and northwestern Kenya, where there is a clear gradient in aridity, from less dry in the West to drier in the East and less dry in the South to drier in the North. The study included four study sites, one agro-pastoral and one pastoral in each country (Table 1, Figure 1). The sites were established in 2020 as part of the Drylands Transform project, in a participatory approach involving community elders, local administration, extension officers and researchers. Sites were selected based on cultural and migration cross-border interactions and proximity to Livestock Cafés (Dryland Transform’s sites for rangeland restoration trials and knowledge sharing hubs). The proximity of a health facility and the security situation were also considered when choosing a site.

**Table 1.** Study sites and their traditional dominant livelihoods.

	Kenya	Uganda
Agro-pastoral sites	**Chepareria** (West Pokot County, Kipkomo sub-county)	**Matany** (Napak District)
Population	57,787 (Chepareria)	163,600 (Napak)
Pastoral sites	**Lokiriama** (Turkana County, Loima sub-county)	**Rupa** (Moroto District)
Population	107,795 (Loima sub-county)	123,800 (Moroto)

Sources [23,24].

#### Kenya

##### Chepareria

The site of Chepareria in West Pokot County is the southernmost location of this study. The average annual temperature in West Pokot ranges from 15°C to 30°C in the highlands and 24°C to 38°C in the lowlands [25]. The area has a bimodal rainfall pattern, with rainy periods from March to May and August to November. Mean annual rainfall is 600 mm, varying by altitude. The main livelihood activities are agro-pastoralism in the lower altitude areas and mixed farming in the upper areas [26].

##### Lokiriama

The northernmost site, Lokiriama in Turkana County, is characterized by an arid to semi-arid climate, with temperatures ranging between 20°C and 41°C with a mean of 30.5°C and annual rainfall between 52 mm and 480 mm (average 200 mm) [27]. The population of Lokiriama depends predominantly on pastoralism and a nomadic lifestyle relying on livestock such as camels, goats, and a small percentage on cattle. Some households also practice trading, small business, mining and seasonal subsistence farming.

#### Uganda

The two Ugandan study sites share a semi-arid climate with a dry season from November to March, and a rainy season from April to August with an average annual rainfall of 800 mm (range 300–1200 mm). Temperatures range from 15°C to 33°C, with January and February being the hottest months [28].

##### Matany

Matany was selected as the study site within the district of Napak. Based on the latest livelihoods’ mapping of Karamoja, Napak has been associated with two main livelihood zones including the Central Sorghum and Livestock Livelihood Zone in northern Napak and the mixed crop zone in southern and central Napak [29].

##### Rupa

For Moroto, Rupa was chosen as the northern study site. Moroto District is located in north-eastern Uganda at the border to Turkana, Kenya, and part of the Central Sorghum and Livestock Livelihood Zone [30]. Traditionally, this region was characterized by pastoral livelihoods, but today agro-pastoral forms are dominating, with livestock (cattle, goats, sheep) playing a more crucial role than crops. Rain-fed agriculture is common, although the production is insufficient due unreliable rainfall. Additionally, households participate in various economic activities (selling firewood and charcoal, agricultural labor and brewing) [30].

### Sampling approach and recruitment

#### Sample size determination

The survey’s sample size was calculated separately for the four study sites, aiming at similar characteristics as the target population, enabling generalization of findings in each of the four study sites [31]. The sample size was calculated based on anthropometric data, using Global Acute Malnutrition (GAM) as the main outcome of interest of the study. Including the prevalence of GAM, precision, design effect, average household size, proportion of children under 5 years, and non-response rate, the Emergency Nutrition Assessment for SMART software [32] was used to estimate each site’s sample size. For this estimation, we used location-specific data (household size, number of children under 5 years per household and prevalence of GAM) from the Smart Nutrition Surveys 2019 for West Pokot County [25] and for Turkana [33], respectively. For Napak (study site Matany) and Moroto (study site Rupa), data were extracted from the Integrated Food Security Phase Classification (IPC) report of 2021 for Karamoja [34]. Based on these estimates, the total required household sample size was 920 (213 in Chepareria, 200 in Lokiriama, 226 in Matany and 281 in Rupa).

#### Sampling procedure

In the Kenyan sites, village or household registers were inaccurate or absent; therefore, systematic random sampling was performed for the selection of households. Using the livestock cafés of the Drylands Transform project as a starting point, villages were randomly sampled radially around the livestock café at a radius of 1–15 km. Within each village, households were randomly picked in all four directions. Due to security issues, western villages in Lokiriama were not selected.

In Uganda, reliable registers were available. Therefore, a two-stage cluster random sampling procedure was used. In the first stage of village selection, a list of all parishes and their estimated number of residential households in each site was obtained. Using a probability proportional to population size of the parish, the number of villages required per parish per site was determined based on the number of households in the parish. From all listed villages in each parish, the required number of villages was randomly selected using https://www.random.org/lists/. See Appendix for details of the sampling procedure.

### Standardized interview and anthropometric measurements

#### Interview tool

The household survey included 10 sections (Table 2). The aim was to interview both a male (usually, the household head) and a female respondent and to include one index child from each household. The child should be the youngest child aged 6–59 months, and the female respondent should preferably be the mother or caretaker of that child. If there was no child, a pregnant or lactating woman should be chosen if available.

**Table 2.** Main content of the 10 sections of the household survey.

*Male and female respondent*		
1	Sociodemographics	Education, marital status, activities, social networks
*Male respondent*		
2	Livelihoods	Land access and ownership, livestock, crops and trees
3	Migration	Seasonal livestock migration, human migration
4	Assets and wealth	Ownership of assets, access to water sources
5	Finances	Income, expenditures, future perspectives
6	Shocks and conflicts	Livestock-related shocks, conflicts in communities and family, climate change perceptions
*Female respondent*		
7	Social networks and female work	Services for the household, female respondent’s income generating activities, kitchen garden, future perspectives
8	Shocks and conflicts	Crops-related shocks, conflicts in community and family, climate change perceptions
9	Nutrition	Household Food Insecurity Access Scale (HFIAS) [35], Months of Adequate Household Food Provisioning (MAHFP) questionnaire [36], list-based 24-h recall of food intake of index child [37] & index woman [38]
10	Health and anthropometry	Diseases and deaths, vaccination of index child; weight, length/height, mid-upper arm circumference (MUAC) of index child & index woman [39]

#### Anthropometry

We measured the selected index child’s body length (children aged 6–23 months) or body height (children 24–59 months), weight and mid-upper arm circumference (MUAC). For the selected index woman, body height, weight and MUAC were taken. The equipment used for measuring anthropometry included a mobile length measuring board for children aged 6–23 months (Seca 417), mobile height measurement for older children and adults (Seca 213), a scale for mother/child function (Seca 874 dr), and standard MUAC-tapes [39].

#### Testing and enumerator training

Household survey data were collected using the Open Data Kit (ODK) application [40].

A pretest of the survey tool was conducted close to the Rupa site, Uganda, February 2022, followed by 6-day training of the data collection teams in May. For each study site, a team leader and four enumerators were recruited. Team leaders were PhD students or project assistants of Drylands Transform; enumerators were recruited locally and spoke the local language. The enumerator training involved getting acquainted with the interview questions and discussing suitable translations in the relevant local languages. It was ensured that all team members understood the questions and possible responses in the same way.

### Data collection

Two project assistants based in Moroto (for the Ugandan sites in Rupa and Matany) and West Pokot (for the Kenyan sites in Chepareria and Lokiriama), respectively, supported the team in identifying and contacting local leaders at the sites. These local leaders accompanied the team when accessing the field, supported in recruiting participants, and helped to identify the sampled villages.

Interviews for the baseline survey were conducted in the selected households in late May and June 2022. Between February and March 2023, a follow-up survey was conducted with the same households. The two surveys were planned to target a wet and a dry season, respectively, but due to failing rains, the baseline was conducted at the end of the dry season, with little rain starting. This can be considered the ‘lean season’ when resources have declined, and land preparation or crop planting is ongoing.

### Ethical considerations

Ethical permits were obtained in all three involved countries (Kenya [P721/09/2021], Uganda [CAES-REC-2023-1], Sweden [2021-05,780-01]) and have been updated yearly in Kenya and Uganda in line with local requirements. Data collection followed ethical guidelines ensuring each respondent gave their written consent for themselves and for their child (either with a signature or thumb print) before the interview. The enumerators also ensured confidential handling of the participant data. Quality control was enhanced through training of enumerators, pilot testing and implementation of quality checks during the data collection process. Data management secure storage was paid for (GitHub organization account), and protocols of data management, cleaning and sharing were followed to maintain data integrity.

### Statistical methods

#### Quantification of variables

The standardized Household Food Insecurity Access Scale (HFIAS) generates four distinct categories of food insecurity, based on the availability or lack of food in the previous 4 weeks [35]. We present prevalences of the two most severe categories, moderate and severe food insecurity. The Months of Adequate Household Food Provisioning (MAHFP) questionnaire quantifies the number of months with adequate food access in the past 12 months [36]. Nutritional status of the index child was based on MUAC and categorized malnourishment as severe, acute (<11.5 cm), moderate, acute (11.5–12.4 cm), at risk (12.5–13.5 cm) and healthy (>13.5 cm) in line with UNICEF standard [39].

Malnutrition of the index woman was defined based on body mass index (BMI), rather than on MUAC, because arm circumference was found to be unsuitable due to the women´s heavy physical workload. Women with a BMI < 18.5 were categorized as undernourished.

#### Statistical analyses

For categorical variables, absolute and relative frequencies for the whole study population and per study site were calculated. Normally distributed continuous variables were presented with mean and standard deviation; for skewed variables, we chose the median and either minimum and maximum values (variable household size), the 5th/95th, or 25th/75th percentiles. The 5th/95th percentiles were chosen for variables capturing distance to the nearest water source, to highlight the extremes that some of the households in drylands must cope with. The variation of all other continuous variables was described using the 25th/75th percentiles. Due to the descriptive nature of this paper, no statistical tests were performed.

All data analyses were done using the Stata software (Version 18, Stata Corp, 2023).

## Results

### Study population

For all study sites, the final household sample size came very close (96–99%) to the numbers required by the power calculations. Households were predominantly male-headed (90.4%) and had on average six members and a maximum of 15 (Table 3). The majority (80.4%) had at least one child aged 6–59 months (index child); ranging from 67.4% in Matany to 97.5% in Lokiriama. Nearly all households in Kenya owned livestock, compared to just over half in the Ugandan sites. Expenditures exceeded income for virtually all households at least during some months each year; the overall median 5 months.

**Table 3.** Study population: household characteristics and livelihoods at the four study sites.

		Kenya		Uganda	
	Total	Chepareria	Lokiriama	Matany	Rupa
**Total** N (%)	944 (100)	222 (23.5)	202 (21.4)	230 (24.4)	290 (30.7)
**HH Male headed** N (%)	852 (90.4)	214 (96.8)	176 (87.1)	182 (79.1)	280 (96.6)
**HH size** Median (min-max)	6 (1–15)	6 (2–15)	6 (3–12)	5 (1–13)	5 (2–14)
**Child 6–59 months**^1^ N (%)	759 (80.4)	176 (79.3)	197 (97.5)	155 (67.4)	231 (79.7)
**Livestock ownership** N (%)					
Current	705 (74.7)	216 (97.3)	197 (97.5)	132 (57.4)	160 (55.2)
Previous	205 (21.7)	3 (1.4)	4 (2.0)	82 (35.7)	116 (40.0)
Never	34 (3.6)	3 (1.4)	1 (0.5)	16 (7.0)	14 (4.8)
**Expenditures exceeding income, months/year** Median (p25; p75)	5 (3; 7)	5 (4; 7)	6 (4; 7)	6 (4; 7)	2 (1; 6)
**Main livelihood source** N (%)					
Livestock	168 (17.8)	10 (4.5)	142 (70.3)	3 (1.3)	13 (4.5)
Crops	158 (16.7)	6 (2.7)	2 (1.0)	112 (48.7)	38 (13.1)
Both livestock/crops	426 (45.1)	199 (89.6)	38 (18.8)	101 (43.9)	88 (30.3)
Other source	192 (20.3)	7 (3.2)	20 (9.9)	14 (6.1)	151 (52.1)
**Kitchen garden**^2^ N (%)	352 (37.3)	83 (37.4)	48 (23.8)	36 (15.6)	185 (63.8)
**Access to communal grazing land** N (%)	645 (68.3)	30 (13.5)	201 (99.5)	167 (72.6)	247 (85.2)
**Seasonal livestock migration**^3^ N (%)	389 (55.2)	43 (19.9)	137 (69.5)	94 (71.2)	115 (71.9)
**Distance (km) to borehole for drinking water** Median (p5; p95)	0.5 (0.1; 4.0)	1.0 (0.2; 4.0)	1.0 (0.5; 6.0)	1.0 (1; 6.0)	0.2 (0.0; 1.0)
**Distance (km) to borehole for livestock water** Median (p5; p95)	0.8 (0.1; 7.0)	0.8 (0.0; 1.5)	1.8 (0.5; 10.0)	0.6 (0.1; 5.0)	0.5 (0.1; 6.0)

HH=Household, N=number, p5=5th percentile, p25=25^th^ percentile, p75=75th percentile, p95=95th percentile.

^1^Presence of index child; youngest child aged 6–59 months in household

^2^Defined as a garden of the female respondent, where vegetables or fruits are grown.

^3^Among livestock owning households.

Most of the households, on average 80%, had their main income from livestock or crop farming. Livestock were important livelihood sources in all sites, but while 70.3% in Lokiriama relied solely on animal husbandry, this was rarely the case at the other three sites. At the traditionally pastoralist site of Rupa, 30.3% of households combined crops and livestock, but half of the households had other main livelihood sources than either crops or livestock – most common charcoal burning or selling, casual work, mining, producing or selling brew (data not shown). Kitchen gardens were also more common in Rupa than in the other sites (63.8%, compared to 15.6–37.4% in the other sites).

At all sites except Chepareria, the majority (72.6–99.5%) of the households had access to communal grazing land, and seasonal livestock migration was common.

Generally, the distance to water sources was large. Most of the households had to walk at least 1 km to get water for human consumption from a borehole, often the closest water source. The situation was better in Rupa, where most had drinking water within a few hundred meters. Drinking water for livestock was usually further away.

### Challenges for the households

In the traditional pastoralist areas of Lokiriama and Rupa, about 43% of the households reported human migration during the 6 months preceding the survey, mostly only once (Table 4). In Lokiriama, the main reasons for human migration were migrating with livestock (named as reason by 88.2% of migrating households) and drought (89.4%), while in Rupa 41.5% of migration was with livestock; and almost one quarter to find work.

**Table 4.** Challenges of households at the four study sites.

	Total	Kenya		Uganda	
		Chepareria	Lokiriama	Matany	Rupa
	944 (100%)	222	202	230	290
**Human migration in last 6 months** N (%)	272 (28.8)	16 (7.2)	85 (42.1)	41 (17.8)	130 (44.8)
**Main reasons of first migration** N (%)					
Livestock migration	132 (48.5)	2 (12.5)	75 (88.2)	1 (2.4)	54 (41.5)
Drought	91 (33.5)	0 (0.0)	76 (89.4)	0 (0.0)	15 (11.5)
Work	61 (22.4)	3 (18.8)	2 (2.4)	25 (61.0)	31 (23.9)
Food shortage	39 (14.3)	0 (0.0)	16 (18.2)	10 (24.4)	13 (10.0)
**Abandonment of livestock keeping (among ever-owners)** N (%)	205 (22.5)	3 (1.4)	4 (2.0)	82 (38.3)	116 (42.0)
**Reason for livestock abandonment** N (%)					
Drought	23 (11.2)	2 (66.7)	3 (75.0)	3 (3.7)	15 (12.9)
Theft/raids	165 (80.5)	0 (0.0)	1 (25.0)	56 (68.3)	108 (93.1)
Poverty	25 (12.2)	1 (33.3)	2 (50.0)	9 (11.0)	12 (11.2)
Security	38 (18.5)	0 (0.0)	0 (0.0)	3 (3.7)	35 (30.2)
Animal diseases	113 (55.1)	3 (100.0)	2 (50.0)	31 (37.8)	77 (66.4)
**Climate change perceptions, male respondent**					
**Climate has changed** N (%)	903 (96.1)	207 (94.5)	202 (100)	225 (97.8)	269 (93.1)
**Type of perceived climate change** N (%)					
Drier	469 (51.9)	132 (63.8)	110 (54.5)	110 (48.9)	117 (43.5)
Hotter	377 (41.8)	106 (51.2)	88 (43.6)	43 (19.1)	140 (52.0)
Unpredictable	365 (40.4)	128 (61.8)	64 (31.7)	64 (28.4)	109 (40.5)
Shorter rain season	397 (44.0)	89 (43.0)	113 (55.9)	78 (34.7)	117 (43.5)
**Worried much or extremely about climate change** N (%)	758 (83.7)	196 (93.3)	189 (93.6)	151 (67.1)	222 (82.5)
**Shocks in the last 6 months** N (%)					
Livestock deaths	497 (70.5)	124 (57.4)	161 (81.7)	72 (54.6)	140 (87.5)
Livestock raids	257 (36.5)	0 (0.0)	61 (31.0)	47 (35.6)	149 (93.1)
Harvest reduction	449 (69.1)	139 (66.5)	11 (52.4)	124 (70.5)	175 (71.7)
**Conflicts** N (%)					
Between communities	346 (36.7)	1 (0.5)	76 (37.6)	88 (38.3)	181 (62.4)
Within the community	183 (19.4)	34 (15.3)	12 (5.9)	33 (14.4)	104 (35.9)
Within the family (male respondent)	206 (21.8)	17 (7.7)	8 (4.0)	82 (35.7)	99 (34.1)
Within the family (female respondent)	219 (23.2)	21 (9.5)	25 (12.4)	96 (41.7)	77 (26.6)
**Food insecurity**					
Moderate food insecurity (HFIAS) N (%)	151 (16.6)	84 (44.9)	3 (1.5)	28 (12.2)	36 (12.4)
Severe food insecurity (HFIAS) N (%)	724 (79.7)	72 (38.5)	199 (98.5)	200 (87.3)	253 (87.2)
Months of adequate food (MAHFP) Median (p25; p75)	4 (3; 6)	5 (4; 8)	4 (2; 5)	5 (4; 6)	3 (2; 5)
**Undernutrition**					
Index child (MUAC < 12.5cm) N (%)	83 (11.1)	6 (3.4)	21 (10.8)	17 (11.3)	39 (17.0)
Index woman^1^ (BMI < 18.5) N (%)	385 (42.5)	59 (26.8)	59 (33.5)	120 (53.6)	147 (51.6)
**Poor/very poor subjective health, female respondent** N (%)	78 (8.3)	11 (5.0)	41 (20.3)	9 (3.9)	17 (5.9)
**Food aid** (last 24h)					
Feeding program^2^ - Index child	68 (7.3)	1 (0.5)	57 (28.2)	1 (0.4)	9 (3.1)
Relief food^3^ - Index child	69 (7.4)	0 (0)	66 (32.7)	1 (0.4)	2 (0.7)
Feeding program^3^ - Index woman	34 (3.6)	1 (0.5)	30 (14.9)	1 (0.4)	2 (0.7)
Relief food^3^ - Index woman	108 (11.5)	1 (0.5)	104 (51.5)	0 (0)	3 (1.0)

N=number, p5=5th percentile, p25=25th percentile, p75=75th percentile, p95=95th percentile.

^1^Female weight included traditional necklaces if worn.

^2^Feeding program: Has eaten food from Supplementary Feeding Program by WHO/UNICEF/WFP targeting undernourished children.

^3^Relief food: Has eaten relief food, provided free of charge by governmental or other organizations.

At the two Ugandan sites, a large proportion of previous livestock owners (38.3% in Matany and 42.0% in Rupa) had completely abandoned livestock keeping as a livelihood form. The most common reasons in both sites were theft/raids (livestock grabbing) and animal diseases; in Rupa, security reasons (fear of attacks or violent conflicts) were also common. In Kenya, only a few households had given up livestock keeping.

Most participants agreed that the climate had changed, becoming drier, hotter, more unpredictable and that the rainy seasons had become shorter. The majority worried much or extremely much about this, especially in the Kenyan sites.

In the last 6 months, on average, 70% of households had been affected by livestock deaths (highest in the pastoral sites, Rupa and Lokiriama) and harvest reductions (highest in the Ugandan sites). Almost all households in the Rupa site had experienced livestock raids, forcing them to abandon their permanent homesteads. Conflicts were generally more often reported in the Ugandan sites than in Kenya. Rupa was particularly affected by conflicts between communities (62.4%) and within communities (35.9%).

Food insecurity was overwhelmingly common in all sites, with nearly 80% households severely and 16.6% moderately food insecure during the month prior to the survey, as measured by HFIAS. The Kenyan sites had the largest variation, with Chepareria best off with 38.5% severely food insecure, compared to 98.5% in Lokiriama. Households had an overall median of 4 months with adequate food available (MAHFP scale) during the preceding year.

Despite the high level of food insecurity, the prevalence of severe and moderate undernutrition among selected index children was relatively low with an average of 11.1%, ranging from 3.4% in Chepareria to 17% in Rupa. Undernutrition was higher among the women, with one-third of the Kenyan index women and more than half of Ugandan women being underweight. On the other hand, in three of the sites, only a few responding women rated their subjective health as poor or very poor, except for Lokiriama where the prevalence was 20.3%.

Unlike at other sites, in Lokiriama, there was a high presence of food aid; during the 24 h prior to the interview, around 30% of the children had eaten foods from supplementary feeding programs by WHO/UNICEF/WFP for undernourished children or relief foods (about half had eaten both types). More than half of the women had eaten relief food, and 14.9% reported that they had also eaten food from the supplementary feeding program.

## Discussion

The Karamoja border region of Uganda and Kenya is a rural setting, historically characterized by pastoralism and seasonal livestock migration. Due to climatic, environmental and societal changes, many communities are today in a transition to new livelihood patterns. Such profound changes necessitate a transformation for sustainable development.

The Drylands Transform project [19, 21] aims at providing communities and decision makers with insights for a sustainable transformation in this region. The present paper described the methodology of the first of two household surveys, as well as household characteristics and human wellbeing.

### Livelihoods and conflicts

At all sites, livestock is still an important source of income; only a minority of households had never owned livestock. However, pastoralism was the main livelihood only in Lokiriama, northern Kenya, the driest and most remote location. At the same time, Watete et al. observed that most pastoralists in northern Kenya depend largely on other sources of income [41]. It is remarkable that more than one out of three households at the Ugandan sites had given up livestock due to external threats, including insecurity and raids. This alludes to changing livelihood options and diversifications currently reported in the area [42]. Violent conflicts in the form of livestock raids were named by more than 90% of respondents in Rupa, the site with the highest proportion of families giving up livestock keeping. Also, other forms of inter- and intra-community conflicts were more common than at the other sites. Thus, conflicts are a substantial barrier to wellbeing and sustainable development, particularly affecting Rupa. What used to be a pastoral society, dependent on livestock, is currently resorting to other sources of income.

The decline of transhumance pastoralism reflects a transition to more sedentary livelihoods based on agriculture and off-farm work in the informal sector such as mining, brewing and charcoal selling [13], which also was observed in our study sites. Due to conflicts and other drivers, income diversification through wage income and self-employed work in the informal sector have increased over the last years in Karamoja [13]. However, according to a study from northern Kenya, off-farm work is usually less profitable compared to pastoralist or agro-pastoralist livelihoods, and hence it is often done by poorer, younger people lacking land or livestock assets [41]. Furthermore, for male breadwinners, livestock loss and lack of viable alternative income sources restricts, their ability to provide for their families. This has the potential to affect traditional gender roles and to incite domestic tension [43] and might explain the higher prevalence of intra-family conflicts at our Ugandan sites, as reported by both male and female respondents. A qualitative study from northeastern Uganda showed a decrease in men’s ability to fulfill their traditional provider roles, and consequently, an increase in women’s off-farm work to supplement household income, playing dual roles as caregivers and income earners [44].

### Water

Given the semi-arid climate of the Karamoja region, access to water is a key obstacle of development for a rural population dependent on timely rainfall for pasture and agricultural production. Our study sites receive 200–1200 mm rain per year; inter-annual variations of rainfall are large and appear to be increasing due to climate change [45,46]. In such dry regions, fetching water is usually a large burden for households. For some households at our site in Lokiriama, the nearest source for potable water was more than 6 km away, requiring women to spend many hours daily collecting water. As Carr et al. [47] stated, ‘time poverty’ limits other activities and the improvement of overall wellbeing of women and households in water scarce settings.

Anthropogenic climate change and variability cause rainfall in many regions to become less reliable and droughts more common. In the last decades, the short rainy season has become wetter, while levels of precipitation and the duration of the long rains have decreased. However, apart from an increase in hot days, this region is projected to experience higher levels of annual rainfall (the ‘east African rainfall paradox’) during this century, and droughts are not expected to worsen [48].

At the same time, most of our respondents reported being worried about climate change, many perceiving shorter rainy seasons and a drier and hotter climate, in line with scientific observations. If this trend continues, there is a risk that water sources will cease for increasingly longer periods during the dry season, making distances even longer, and reducing the availability of water for human consumption, agriculture and livestock. Carr et al. [47] predict a profound increase in time for water collection due to higher temperatures and/or a decrease in precipitation for some regions, albeit not for Kenya and Uganda, the setting of our study. Generally, increased heat stress will lead to a higher demand for water for livestock but will at the same time reduce meat and dairy production. Heat and changing precipitation patterns, including prolonged droughts, followed by heavy rainfall, have the potential to aggravate morbidity and mortality of both humans and livestock [49]. Water scarcity, as Huynh and Resurreccion [50] pointed out, intersects with and is determined by other inequities (gender, low social status and age), hence contributing to vulnerability of women and households in disadvantaged communities. There is thus an urgent need to provide reliable access to safe drinking water and to enhance agricultural production methods less dependent on reliable rainfall.

### Malnutrition and food insecurity

Undernutrition, aside from child morbidity and mortality, is a key indicator of population development and wellbeing [51–54]. In our study population, the prevalence of malnutrition in children aged 6–59 months varied substantially between sites. Surprisingly, Lokiriama, the most remote and socio-economically disadvantaged, and by far driest study site, faced lower levels of child and female undernutrition than Rupa just across the border, the site with the highest prevalence. Nevertheless, food insecurity was widespread at all sites, with prevalences being lower only in Chepareria. While food shortage in Lokiriama was compensated by food aid through child feeding programs or the delivery of food aid, this was rarely reported by Ugandan women in our study. The uneven external support, despite similar deficiencies, might explain higher prevalences of malnutrition in Rupa than in Lokiriama. Data from local governments or organizations should verify these assumptions. Feeding programs have been present in the Karamoja region for a long time [55], however, reportedly a large proportion of malnourished children were not enrolled in these [56]. It is also important to note that dietary diversity in our study population is overall low, showing widespread hidden hunger aside from shortage of food. Further quantitative analyses of our project will highlight seasonal variability and drivers of dietary diversity, acute and chronic malnutrition at the household and community level. Recently, we have explored community experiences regarding maternal employment and its implications for child nutrition and health in northeastern Uganda [44].

Generally, undernutrition appeared much higher in women than in children, especially in Uganda where more than half of the examined women were rated as undernourished. Prevalences among children and women, however, cannot be directly compared, since they were based on different measures. We chose BMI as an indicator for malnutrition for women with a cut-off at 18.5, instead of MUAC (as for children). MUAC is a less reliable measure for adult women who engage in hard physical labor, e.g. by carrying water daily over very long distances. Based on a MUAC cut-off 23 cm, 75% of all measured women would have been rated healthy (data not shown).

Interestingly, one out of seven women in Chepareria were overweight or obese (BMI 25 or higher; data not shown), while this was rare in the other sites. This indicates an ongoing epidemiological transition from diseases of poverty to diseases of affluence in the most advantaged site. It also hints at the co-existence of over- and undernutrition within the same community or even household, a so-called double burden of malnutrition, which occurs typically in rapidly changing settings in low- and middle-income countries [57,58].

### Kitchen gardens

Kitchen gardens have been shown to impact positively on food security and dietary diversity in many geographical regions [59], although not in all [60], and more studies are needed to better understand the prerequisites in different settings. About one-third of households in the present study maintained a kitchen garden, although these might vary in size and character. Kitchen gardens can be a valuable source of nutrients such as fiber, vitamins, minerals and protein, which are currently insufficient in the typically starchy Karamojan diet. Women taking control of kitchen gardens have the potential to become powerful agents of change in Karamoja, combating food insecurity and low dietary diversity. The Drylands Transform project’s livestock cafés are experimental sites for methods of fodder production and land restoration, but also for kitchen gardens [61,62], and they function as knowledge hubs where residents can practice and share new forms of agricultural production. Training-of-trainers workshops for kitchen gardening target particularly women, enhancing female empowerment. As access to water sources is insufficient in most places, kitchen gardens are solely reliant on seasonal rain, which necessitates the promotion of drought-resistant plants and farming methods. Results of the livestock café experiments so far are promising, showing success in growing vegetables, legumes and fruits even when the seasonal rains have been insufficient. Improved kitchen gardens therefore have the potential to tackle some of the challenges described above related to water scarcity, food insecurity, low dietary diversity and malnutrition and can contribute to income generation for rural women.

### Policy and development

The governments of Kenya and Uganda and several non-governmental organizations carry out interventions to help communities cope with extreme shocks. These initiatives include climate change adaptation, water resource management, infrastructure development, conflict resolution, sustainable agriculture and livelihood diversification, aside from general improvements in education and health care. However, some literature argues that the Karamoja border region still faces marginalization and limited government support [63–66]. There are also worries that overreliance on aid could deter the self-initiated traditional efforts to get out of poverty by the locals, affecting their resilience. Therefore, active involvement of communities in planning and conducting interventions is needed.

In Karamoja, as in many other dryland regions, historical pastoralist livelihoods are changing fast [67], although drivers and variations of this change are today not well understood. In response to manifold challenges, income diversification is imperative to foster climate resilience, environmental sustainability, and overall economic and social wellbeing of this vulnerable population.

### Study strengths and limitations

This is, to our knowledge, the first comprehensive, household-based survey conducted in the Karamoja region, with four neighboring, but socio-economically and climatically diverse study sites. In total, 944 households were included, with high response rates even in hard-to-access, remote communities. The interviews included both male and female representatives of each household and covered many topics relevant for pastoralist settings that are usually not addressed in surveys of larger geographical areas, such as the Demographic and Health Surveys [68].

For some topics (household assets, nutrition, food insecurity, anthropometry), we used standardized tools which allow comparison with other studies. Other components were formed to the specific setting in the Karamoja border region. The teams’ knowledge of local customs and language, enumerator training and pretests facilitated improvement in the methods and access to the communities. Familiarity with the setting and the involvement of local leaders also facilitated trust building with respondents. Therefore, we are confident that survey answers are reliable and comparable across sites.

There are, however, some limitations this study faced: Diet and anthropometry were assessed for only one index child aged 6–59 months and one index woman in each household. It would have been valuable to measure these also for older children and males, to detect potential differences in malnutrition within one family. The body weight of women was not corrected for necklaces, which is common particularly at the Lokiriama site in northern Kenya. We assume that only a few women were wearing many heavy necklaces, but a site-specific bias of BMI measures cannot be excluded, causing a potential underestimation of undernutrition. Although enumerators were well trained and supervised by team leaders, and interviews were standardized, the interview situation was difficult to control. Often, privacy could not be guaranteed, which might produce a response bias especially for questions perceived as sensitive, such as livestock herd size, violent conflicts and income. In some cases, it was not clear which of the two interviewees had answered questions of the male or female sections, respectively, causing a risk of bias.

## Conclusions

The two household surveys of the Drylands Transform study population, including more than 900 households, were conducted in the Karamoja border region of Kenya and Uganda. They provide a valuable, small-scale knowledge base for understanding vulnerabilities, opportunities and their determinants in East African pastoral and agro-pastoral communities and beyond. In this paper, we presented the methodology of the baseline study and described household characteristics and challenges for improved livelihoods and wellbeing.

The UN Agenda 2030 has the aim of promoting sustainable development, leaving no one behind [69]. Marginalized drylands like Karamoja need an integrated, holistic approach for development and a systems-based, sustainable transformation of land, livelihoods and society. Transdisciplinary projects like Drylands Transform can contribute to a sustainable transformation of these disadvantaged settings through their close interaction with communities and local and regional stakeholders [21].

Our study identified numerous challenges to human wellbeing, including scant livelihoods, widespread malnutrition, climate change, shocks and conflicts. It also showed partly substantial variations between the four sites of some of these challenges, highlighting potentials for future interventions. A systemic transformation for sustainable development is needed: Increase agricultural productivity aside from the provision of alternative income sources that benefit the community; tackle drivers of conflicts at different levels; secure food security by improved food production through initiatives such as kitchen gardens and income generating activities for both men and women.

Ongoing studies based on the two Drylands Transform surveys will investigate seasonality and other factors contributing to variability in malnutrition, conflicts and shocks, as well as their implications for human wellbeing in the Karamoja border region.

## Supplementary Material

Supplemental Material
