# Supplementary material for: Livelihood dynamics and challenges to wellbeing in the drylands of rural East Africa – the Drylands Transform study population in the Karamoja border region
Source: Glob Health Action. 2025 Apr 24;18(1):2490330. doi: 10.1080/16549716.2025.2490330 (PMC12024496; doi:10.1080/16549716.2025.2490330)
Supplement: Supplemental Material [file ZGHA_A_2490330_SM4908.docx]

# Appendix

### Details: Sampling of households

**Sample size determination**

The sample size of the household survey was calculated in order to generate a sufficiently large, representative sample. This sample should have the same characteristics as the target population, enabling generalization of findings to the target population [1]. For the Drylands Transform project, the target population is the population in the four sites (Rupa and Matany in Uganda; Lokiriama and Chepareria in Kenya), representing the pastoral and agro-pastoral systems in the two countries. The sample size for the survey should therefore be large enough to ensure that findings from the sample are generalizable to the population in each of the four study sites.

The sample size was calculated based on anthropometric data, focusing on child malnutrition, one of the main outcomes of interest of the study. For each household, one index child was selected.

The Emergency Nutrition Assessment for SMART software [2] was used to estimate each location’s sample size as follows:

$$n=t^{2}*\frac{p\left( 1-p \right)}{d^{2}}*DEFF (1)$$

Where *n* is the sample size for children, *d* is the desired precision, *t^2^* is the t value linked to the 95% confidence interval for cluster sampling, *p* is the estimated proportion of a nutritional indicator under study, *DEFF* is the design effect. Precision is a measure of the consistency of the survey results. A precision of 5% indicates that the true population value is within a range 5% higher or 5% lower than the estimated value. The small range of +-5% represents a higher precision and lower margin of error. The nutritional indicator *p* in our case was the prevalence of Global Acute Malnutrition (GAM). The design effect (DEFF) is a correction factor to account for the heterogeneity of acute malnutrition between clusters (in our case, villages). If the prevalence of malnutrition in a few villages is higher than the rest of the villages, it will not be representative of the whole survey area and thereby overestimate the total estimate prevalence estimate. To compensate for this, there is a need to increase the sample size by using a correction factor called design effect, in this study fixed at 1.2.

The estimated values for household size, number of children under 5 years per household and GAM for Matany and Rupa were extracted from the Integrated Food Security Phase Classification (IPC) report of 2021 for Karamoja [3]. The estimates for Lokiriama and Chepareria came from the Smart Nutrition Surveys 2019 for Turkana [4] and West Pokot County [5], respectively.

The formula in Equation (1) gives the estimated number of children at each site. Equation (2) was used to estimate the total number of households required to meet the target number of children, given the average household size and proportion of children under 5 years.

$$Number of households to be visited=\frac{Sample size for children (one per household)}{Average household size x percentage of children>5 x 0.9} (2)$$

Based on these estimates, the total required household sample size was 920. Table 1 shows the sample size estimations for children and households per study site.

**Table A1: Required sample size per site in cluster design**

| **Parameter** | **Chepareria** | **Lokiriama** | **Matany** | **Rupa** | **Total** |
| --- | --- | --- | --- | --- | --- |
| Average household size (n) | 6 | 7.89 | 4 | 4.6 |  |
| Prevalence of Global Acute Malnutrition (%) | 11.7 | 20.2 | 9.4 | 14.2 |  |
| Desired Precision +- (%) | 5 | 5 | 5 | 5 |  |
| Children under 5 years (%) | 19% | 24.3% | 22.1% | 22.1% |  |
| Design Effect | 1.2 | 1.2 | 1.2 | 1.2 |  |
| Non-response Rate (%) | 5% | 5% | 5% | 5% |  |
| Sample size children 6-59 months | 207 | 324 | 171 | 245 | 947 |
| **Required household sample size** | **213** | **200** | **226** | **281** | **920** |

### Sampling procedure

The sampling procedure ensured that each household in the selected villages each had a known, non-zero equal probability of being selected, and that the selection of one household was independent from the selection of another.

The study used a two-stage cluster random sampling procedure. In the first stage, a list of all parishes and their estimated number of residential households in each site was obtained. Using probability proportional to population size of the parish, the number of villages required per parish per site were determined based on the number of households in the parish. From all listed villages in each parish (Uganda) and sub-location (Kenya), respectively, the required number of villages was randomly selected using <https://www.random.org/lists/>. A list of households in each of the selected villages was obtained by the village chairperson; 12-16 households were randomly selected from this list. Those of which neither male nor female household heads were present were replaced with another random household.

In the Kenyan study sites Chepareria (West Pokot) and Lokiriama (Turkana), village or household registers were inaccurate or absent; therefore, systematic random sampling was performed for the selection of households. Using the livestock cafés of the project as a starting point, enumerators moved in all directions to recruit households. Villages were randomly sampled radially around the livestock café at a radius of 1-15 km. Within each village, households were randomly picked in north, south, east and west direction. The first homestead was selected, and the next three omitted, selecting the fourth fifth homestead and so on. If nobody was present in the homestead, the adjacent house was selected instead.

## **References**

1. Bartlett JE, Kotrlik JW, Higgins CC. Organizational research: Determining appropriate sample size in survey research. Information Technology, Learning, and Performance Journal. 2001;19(1)

2. SMART. ENA Software for SMART: ACF. Standardized Monitoring and Assessment of Relief and Transitions Initiative; 2020. http://smartmethodology.org/survey-planning-tools/smart-emergency-nutrition-assessment/. Accessed April 2022.

3. IPC. Uganda: Acute Malnutrition Situation February - July 2021 and Projection for August 2021 - January 2022 (Karamoja). Integrate Food Security Phase Classification Initiative; 2022. https://www.ipcinfo.org/ipc-country-analysis/details-map/en/c/1155315/?iso3=UGA. Accessed April.

4. Turkana County Government. Turkana County SMART Nutrition Surveys - June 2019 Report. Turkana County Government; 2019. http://www.nutritionhealth.or.ke/wp-content/uploads/SMART%20Survey%20Reports/Turkana%20County%20SMART%20Survey%20Report%20-%20June%202019.pdf. Accessed March 2024.

5. West Pokot County Government, West Pokot Multi-Stakeholders' Committee. Integrated Smart Survey West Pokot County - June 2019. West Pokot County Government; 2019. http://www.nutritionhealth.or.ke/wp- content/uploads/SMART%20Survey%20Reports/West%20Pokot%20County%20SMART%20Survey%20Report%20-%20June%202019.pdf. Accessed April 2024
